# Cases from Practice

**Published:** 1892-09

**Authors:** 

**Affiliations:** Hamburg, Germany


### CASES FROM PRACTICE.

### Dr. Hesse, of Hamburg, Germany.

[Translated from *Archiv. für Homœopathie* by A. McNeil, M. D., San Francisco, Cal.]

Asthma.—September 8th, 1889.—Mrs. P. She is of middle age, looks healthy and well nourished; hair black; has complained of attacks of asthma for a long time. Soon after going to sleep she is wakened by difficult respiration; her chest feels as if constricted; must lie with her head high; sit up or get out of bed. In the morning she is covered with sweat.

She has a sensation in the throat as if there was something in it. She also has a peculiar slight pain in the throat which she does not feel when eating. Lachesis$^{30}$, a few pellets to be taken on going to bed.

May 25th, 1891.—She returned because of other complaints. The medicine helped her rapidly. For some weeks now she has had pains extending from the ear to the shoulder and chest; five to eight diarrhœic stools in twenty-four hours, coming both day and night, with rumbling in the bowels and pains around the umbilicus. She has the same sensation in the throat as formerly. All symptoms better in the open air. Pulsat. five powders, to be taken night and morning.

June 11th.—The diarrhœa continued only a short time after the medicine; pain only occasionally; the throat is better. Placebos, with instruction to return if her complaints did not cease entirely in a short time. She did not come back.

The indications were not strong for Pulsat. It with many other drugs has the sensation as if there was something in the throat.

Lachesis has aggravation after sleeping, and, as I have learned in a number of cases, "aggravation on falling asleep."

Psoriasis.—April 2d, 1891.—A young woman has complained for a number of weeks of a dry herpes which first appeared on her face and then extended to her neck and arms. The eruption itched when she became warm in a room. An especial aggravation always occurred when she washed clothes. She has long been subject to hoarseness. She received Phosphorus, a powder to be taken night and morning, for two days.

April 11th.—A considerable improvement. Placebo.

Phosphorus when indicated. The patient cannot bear warmth either internally or externally. (Vomiting when he dips his hands in warm water.) [This symptom is neither in the *Guiding Symptoms* nor in Allen's *Encyclopædia*, therefore I suppose that it is clinical, and may prove valuable.—*Trans.*] It has a particular aggravation from washing clothes—at all events, from working in warm water.

Insanity.—In the beginning of this summer (1891) was called to see a small, thin woman of fifty, suffering from an attack of heat with insanity to which she had been subject since she had a fall nine months ago. These attacks occur suddenly, and continue for hours or days, and then ceased suddenly. Her relatives were undecided whether or not to send her to an asylum for the insane. Her hands and head became hot; she was restless, and would not allow herself to be kept in bed; she was afraid those around her would injure her; she always labored under the delusion that she was not at home, and wanted to go to it; talked incoherently. In her free intervals she was relatively rational, but needed constant watching. The delusion that she is not at home returns so regularly that I turned to the drugs which have this symptom well marked. They are Bell., Bry., Hyos., Lach., and Verat-alb. [Also, Coff. and Opi.] On account of heat of the head I gave Bell., and afterward, because of her confused talking, Lach., but without result. Then Verat., with immediate improvement.

The attacks became less frequent and less violent under the continued use of the remedy in the 30th potency; completely disappeared in ten days. I occasionally see her, as she sometimes complains of heat rising up to her head. Except for this she has been well for months.

I believe Hahnemann says that by the administration of Verat. a part [he says one-third—*Trans.*] of the asylums would be emptied.

